# A Chinese Herbal Decoction, Danggui Buxue Tang, Stimulates Proliferation, Differentiation and Gene Expression of Cultured Osteosarcoma Cells: Genomic Approach to Reveal Specific Gene Activation

**DOI:** 10.1093/ecam/nen085

**Published:** 2011-06-18

**Authors:** Roy C. Y. Choi, Qiu T. Gao, Anna W. H. Cheung, Judy T. T. Zhu, Faye T. C. Lau, Jun Li, Winnie Z. M. Li, Glanice K. Y. Chu, Ran Duan, Jerry K. H. Cheung, An W. Ding, Kui J. Zhao, Tina T. X. Dong, Karl W. K. Tsim

**Affiliations:** ^1^Department of Biology and Center for Chinese Medicine, The Hong Kong University of Science and Technology, Clear Water Bay, Hong Kong; ^2^Jiangsu Key Laboratory for TCM Formulae Research (LTCMF), Nanjing University of Chinese Medicine, Nanjing 210046, China; ^3^Beijing Friendship Hospital, Affiliate of Capital University of Medical Sciences, 95 Yong An Road, Beijing 100050, China

## Abstract

Danggui Buxue Tang (DBT), a Chinese herbal decoction used to treat ailments in women, contains Radix Astragali (Huangqi; RA) and Radix Angelicae Sinensis (Danggui; RAS). When DBT was applied onto cultured MG-63 cells, an increase of cell proliferation and differentiation of MG-63 cell were revealed: both of these effects were significantly higher in DBT than RA or RAS extract. To search for the biological markers that are specifically regulated by DBT, DNA microarray was used to reveal the gene expression profiling of DBT in MG-63 cells as compared to that of RA- or RAS-treated cells. Amongst 883 DBT-regulated genes, 403 of them are specifically regulated by DBT treatment, including CCL-2, CCL-7, CCL-8, and galectin-9. The signaling cascade of this DBT-regulated gene expression was also elucidated in cultured MG-63 cells. The current results reveal the potential usage of this herbal decoction in treating osteoporosis and suggest the uniqueness of Chinese herbal decoction that requires a well-defined formulation. The DBT-regulated genes in the culture could serve as biological responsive markers for quality assurance of the herbal preparation.

## 1. Introduction

Estrogen deficiency is the major cause in developing post-menopausal osteoporosis. Estrogen acts on both osteoblast and osteoclast to inhibit bone breakdown at all stages of life. After menopause, estrogen replacement therapy is an effective treatment for osteoporosis as well as to allay other menopausal symptoms [1]. However, estrogen therapy recently became a subject of debate because clinical studies revealed an increased risk of breast cancer and coronary artery disease in women who take estrogen [2]. In view of these clinical risks, extensive efforts have been devoted to develop different strategies that would yield the benefits of estrogen therapy but with minimal side effects [3]. Herbal medicines, in particular the traditional Chinese medicine, are promising preparations that have fewer side effects [4, 5], and which, indeed, have been used widely for menopausal women as dietary supplements in Asia [6, 7].

Amongst thousands of herbal formulae from traditional Chinese medicine, Danggui Buxue Tang (DBT; a herbal decoction) is a simple combination of two herbs. DBT was first described in *Neiwaishang Bianhuo Lun* by Li Dongyuan in China in AD 1247. Li described the DBT formula should include: 10 *qian* of Radix Astragali (Huangqi; RA), roots of *Astragalus membranaceus* (Fisch.) Bunge or *A. membranaceus* (Fisch.) Bunge var. *mongholicus* (Bunge) P.K. Hsiao, and two *qian* of Radix Angelicae Sinensis (Danggui; RAS), roots of *Angelica sinensis* (Oliv.) Diels. One *qian* equals to about 3 g. In preparing DBT, the mixed herbs were recommended to boil in two bowls of water over a moderate heat until the final volume was reduced by half [8]. Traditionally, DBT has been prescribed to women in China as a remedy for menopausal symptoms. According to Chinese medicinal theory, the daily intake of DBT could raise the “*Qi*” and nourish the “*Blood*” of menopausal women.

Pharmacological results indicated that DBT has the abilities to promote hematopoietic functions: to stimulate cardiovascular circulation; to prevent osteoporosis; to increase anti-oxidation activity, to stimulate immune response and to mimic estrogen effects in the receptor phosphorylation [8, 9]. Besides, RA and RAS are commonly used in treating the age-related diseases, which have been demonstrated in stimulating bone cell proliferation, increasing bone formation and reducing bone re-sorption in patients [10]. By determining the chemical and biological properties of DBT, the optimized conditions of extraction have been established [8, 9], which, interestingly, are in accordance with the weight ratio of 5 : 1 for RA to RAS in the ancient preparation. However, the rationale and action mechanisms for including different herbs in Chinese herbal decoctions had never been fully explained, and more important biological markers are missing in controlling the herbal decoction, which consequently hinders the development of Chinese medicine as disease and disorder remedies. In order to reveal the specific events mediated by DBT in preventing osteoporosis, cell proliferation and differentiation were determined in cultured MG-63 cells (an osteosarcoma cell line). In addition, DNA microarray analysis was used to analyze the gene profiling in MG-63 cells after the treatments of extracts derived from DBT, RA, or RAS. The identified DBT-specific regulated genes in the cell culture could serve as biological responsive markers in quality assurance of DBT and in revealing the action mechanism of this decoction.

## 2. Methods

### 2.1. Plant Materials and Preparation of DBT

Fresh roots were obtained from China in September to October of 2002: 3-year-old *A. membranaceus* var. *mongholicus* from Shanxi and 2-year-old *A. sinensis* from Minxian of Gansu [11, 12] Their corresponding vouchers as forms of whole plants, voucher specimens for *A. membranaceus* var. *mongholicus* and *A. sinensis*, were deposited in the Department of Biology, The Hong Kong University of Science and Technology, China. In preparing DBT, exact amounts of RAS and RA were weighed according to a ratio of 5 : 1 and then mixed well by vortex. The mixture was boiled in 8 volume of water (v/w) for 2 h, and extracted twice; this extraction was shown to be the best extracting condition [8]. RAS or RA alone was extracted by the same method. The extracts were dried by lyophilization and stored at −80°C.

### 2.2. Chemical Standarization of DBT

Ferulic acid was purchased from Sigma (St Louis, MO), calycosin, formononetin and ligustilide (z-isoform) were kindly provided by Prof. Pengfei Tu, Medical College of Peking University; their purities, confirmed by HPLC, were >99.0%. AR and HPLC grade reagents were from Merck (Darmstadt, Germany). The HPLC system consisted of Waters (Milford, MA) 600 pump, 717 auto-sampler and UV/VIS Photodiode Array 2996 Detector were used for all analysis. Chromatographic separations were carried out on a DELTA-PAK C_18_ column (particle size 4.6 *μ*m, 3.9 × 150 mm) with acetonitrile (as Solvent A): 0.01% phosphoric acid (as Solvent B) as mobile phase at a flow rate of 1.0 mL min^−1^ at room temperature. A linear gradient elution was applied from 15 to 65% of Solvent A starting from 0 to 60 min. Samples were filtered through a 0.45 *μ*m Millipore syringe filter unit. A sample of 20 mL was injected for HPLC analysis. The calibration of these chemicals followed previous reports [8, 9].

### 2.3. The Cultures of MG-63 Cells and Rat Osteoblasts

Human osteosarcoma cell line MG-63 was obtained from the American Type Culture Collection (ATCC, Manassas, VA) and was grown in Modified Eagle's medium (MEM), supplemented with 10% fetal bovine serum, 2 mM l-glutamine, 0.1 mM non-essential amino acids, 1 mM sodium pyruvate, 100 U mL^−1^ penicillin, and 100 *μ*g mL^−1^ streptomycin in a humidified CO_2_ (5%) incubator at 37°C. Culture reagents were from Invitrogen (Carlsbad, CA, USA). Before 3 days of the treatment, the medium was changed to MEM-*α* without phenol red containing 2% charcoal-dextran-treated fetal bovine serum. MG-63 cells were seeded onto 12-well plate or 96-well plate in MEM-*α* medium. Next day, the medium was replaced by fresh medium containing 1 mg mL^−1^ DBT, RAS or RA extracts for 48 h. 17*β*-Estradiol (Sigma) dissolved in dimethyl sulfoxide (DMSO) was used as a control. For the control vehicle, 0.0001% DMSO was used. The cell number was determined by a manual cell counting method. In brief, the drug-treated MG-63 cells were detached by treatment with trypsin digestion and resuspended in PBS, and the cell number was counted by a hemocytometer. Besides, a biochemical colorimetric method named 3-(4,5-dimethylthioazol-2-yl)-2,5-diphenyl-tetrazolium bromide (MTT; Sigma) assay was employed. The absorbance at 570 nm was measured using an enzyme-linked immunosorbent assay plate reader (Dynatech MR 5000) [13]. A standard curve of cell number against absorbance at 570 nm was performed. All calibration was done within the linear range of the standard curve. The enzymatic activity of alkaline phosphatase in MG-63 cells was measured by the hydrolysis of *p*-nitrophenyl phosphate as described previously [14]. Briefly, 100 *μ*L of the homogenate derived from the drug-treated MG-63 cells was added to the substrate solution, which contained 10 mM *p*-nitrophenyl phosphate as a substrate in 100 mM diethanolamine buffer (pH 10.5) supplemented with 0.5 mM MgCl_2_. After 30 min of incubation at 37°C, the reaction was terminated by addition of 2 M NaOH, and the activity was determined spectrophotometrically (410 nm) by measuring *p*-nitrophenyl released from the substrate. The enzyme activity was expressed as micromole of substrate cleaved per milligram of cell protein. For the analyses of inhibitors, the cultures were pre-treated with estrogen receptor antagonist ICI 182 780 (0.1 *μ*M; Tocris, Ellisville, MO, USA) and Erk1/2 inhibitor U0126 (10 *μ*M; Sigma) for 3 h before the application of other drugs. Phorbol 12-myristate 13-acetate (0.1 *μ*M; TPA; Sigma) was used as an Erk1/2 activator.

Primary culture of osteoblasts was performed according to Orriss et al. [15]. In brief, calvarias from postnatal 5-day-old rats were collected and undergone sequential enzymatic digestion: 1% trypsin for 20 min, 0.2% collagenase for 20 min and 0.2% collagenase for 40 min at 37°C. After centrifugation at 800 g for 10 min, supernatant containing osteoblastic cells were collected and maintained in DMEM with 10% FBS, 2 mM l-glutamine, 0.1 mM non-essential amino acids, 1 mM sodium pyruvate, 100 U mL^−1^ penicillin, and 100 *μ*g mL^−1^ streptomycin in a humidified CO_2_ (5%) incubator at 37°C. Cell viability was performed by MTT assay as described before. Osteogenic differentiation was induced by the treatment of vitamin C (250 M) and dexamethasone (20 nM), or by the herbal extracts, for 96 h and then subjected to total RNA extraction or alkaline phosphatase assay.

### 2.4. DNA Microarray Analysis

MG-63 cultures were treated 1 mg mL^−1^ RA, RAS or DBT for 24 h to extract the total RNAs by TRIzol reagent (Invitrogen). RNA integrity was confirmed by running the formaldehyde-denaturing gel, with purify of ratio A260/280 > 1.8. Total RNAs were subjected to DNA microarray analysis (Chipscreen Biosciences Ltd., Shenzhen, China) to determine the differential gene expressions in treated MG-63 cells. In brief, cDNAs from control and treatment groups were labeled with Cy5 and Cy3 fluorophores, and hybridized with a DNA microarray chip containing 7458 candidate genes and 384 reference genes. Signals were captured by Generation III array scanner (GE Healthcare, Piscataway, NJ, USA) and data were analyzed by Imagequant 5.0 and Array Vision 6. The significant changes of gene expressions were defined as up-regulation when fluorescent signal in sample was greater than that of control for 200%, or down-regulation when the signal was less than that for 50%.

### 2.5. Estrogen-Activated Promoter Assay

Three repeats of estrogen responsive elements (ERE: 5′-GGT CAC AGT GAC C-3′) was synthesized as described previously [9, 16], and then subcloned into a promoter-reporter vector called pTAL-Luc (Clontech, Mountain View, CA, USA) that has a down stream reporter of firefly *luciferase* gene; this DNA construct was named as pERE-Luc. Cultured MG-63 cells were transfected with pERE-Luc to generate the stable cell line according to a previous report [17]. Serving as controls, two reporters containing three repeats of responsive elements for myocyte enhancing factor 2 (MEF2; 5′-CTA AAA ATA G-3′ in forming pMEF2-Luc) and muscle-regulated transcription factors (MRF; 5′-CAG TTG-3′ in forming pE-box-Luc) were constructed in pTA-Luc. Both MEF2 and E-box are muscle-specific gene activation elements. To determine the estrogenic property, different concentration of estrogen, DBT or other extract were applied onto the cultures for 48 h. Afterward, the medium was aspirated, and MG-63 cells were washed by cold PBS. The cells were lysed by a buffer containing 0.2% Triton X-100, 1 mM dithiothreitol and 100 mM potassium phosphate buffer (pH 7.8) at 4°C. Followed by centrifugation at 14 000 rpm for 4°C 10 min, supernatant was collected and used to perform luciferase assay (Tropix Inc., Bedford, MA, USA); the activity was normalized by equal amount of protein.

### 2.6. Determination of Phosphorylation

The phosphorylation of extracellular signal-regulated kinases (Erk)1/2 was determined by western blot assay. The cultures were serum starved with or without the inhibitors for 3 h before the drug applications. After drug treatments, the cultures were collected immediately in lysis buffer (125 mM Tris-HCl, 2% SDS, 10% glycerol, 200 mM 2-mercaptoethanol, pH 6.8), and the proteins were subjected to SDS-PAGE analysis. Phosphorylated Erk1/2 were recognized by anti-phospho-Erk1/2 antibody (1 : 5000; Cell Signaling, Danvers, MA, USA) at 4°C for 12 h, and horseradish peroxidase (HRP)-conjugated anti-rabbit secondary antibody (1 : 5000; Invitrogen) for 1 h at room temperature. The immuno-complexes were visualized by the enhanced chemiluminescence (ECL) method (GE Healthcare). The band intensities, recognized by the antibodies in the ECL film, in control and agonist-stimulated samples were run on the same gel and under strictly standardized ECL conditions. The bands were compared on an image analyzer, using in each case a calibration plot constructed from a parallel gel with serial dilution of one of those samples: this was to ensure the sub-saturation of the gel exposure.

### 2.7. PCR Analysis

MG-63 cells, or primary cultures of rat osteoblasts, with or without inhibitor pre-treatments were treated with 1 mg mL^−1^ DBT, RA or RAS extract for 12 h. Total RNAs were isolated by TRIzol reagent (Invitrogen), and 5 *μ*g of total RNA was reverse-transcribed by Moloney Murine Leukemia Virus Reverse Transcriptase (Invitrogen) according to the manufacturer's instructions. Qualitative PCR was performed to determine the expression of estrogen receptor (ER) *α* and *β*. The primers were: 5′-TGA AGC ACA AGC GTC AGA GA-3′ and 5′-CGT AGC CAG CAA CAT GTC AA-3′ for ER *α* (501 bp), 5′-CTC TTG GAG AGC TGT TGG AT-3′ and 5′-CTG TGA CCA GAG GGT ACA T-3′ for ER *β* (259 bp), with conditions of 94°C (1 min), 60°C (1 min) and 72°C (1 min) for 30 cycles. The real-time PCR was performed by using SYBR Green Master Mix and Rox reference dye according to the manufacturer's instructions (Applied Bioscience, Foster city, CA, USA). The primers for other transcripts were: 5′-TGT GAT GCC CTT AGA TGT CC-3′ and 5′-GAT AGT CAA GTT CGA CCG TC-3′ for 18S rRNA (320 bp), 5′-TTC ATC ACC ACC ATT CTG GG-3′ and 5′-CAT GGG TCA GCT GGA TGT C-3′ for galectin-9 (289 bp); 5′-AAG GAG GTC TGT GCT GAC-3′ (common chemokine C-C motif; CCL sense primer) and 5′-GAT TCT TGC AAA GAC CCT-3′, or 5′-AGA GAA GGG AGG AGC AT-3′ or 5′-AGG ATG TAT GAC AGA TAG AG-3′ for CCL-2 (242 bp), CCL-7 (356 bp) or CCL-8 (364 bp), respectively. SYBR green signal was detected by Mx3000ptm multiplex quantitative PCR machine (Stratagene, La Jolla, CA, USA), with annealing temperature at 60°C in all cases. Transcript levels were quantified by using the ΔΔ*C*_t_ value method [18]. Calculation was done by using the *C*_t_ value of 18S rRNA to normalize the *C*_t_ value of target gene in each sample to obtain the Δ*C*_t_ value, which then was used to compare among different samples. PCR products were analyzed by gel electrophoresis and melting curve analysis to confirm specific amplifications.

### 2.8. Other Assays

The protein concentrations were measured routinely by Bradford's method with a kit from Bio-Rad Laboratories (Hercules, CA, USA). Statistical tests were made by the Primer program, version 1 (Primer of Biostatistics): differences from basal or control values (as shown in the plots) were classed as significant (*) where *P* < .05, (**) where *P* < .01 and highly significant (***) where *P* < .001 by Student's *t* test.

## 3. Results

### 3.1. The Osteogenic Properties of DBT in Cultured MG-63 Cells

**Figure 1:**
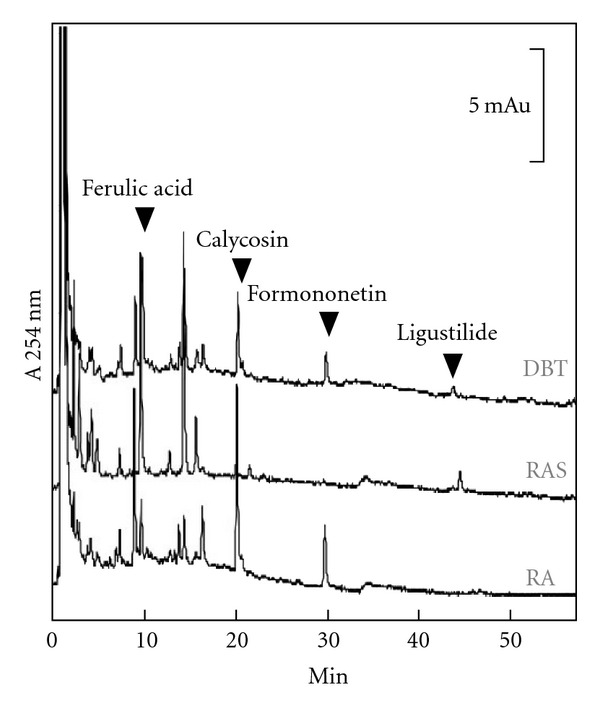
Chemical standardization of DBT by HPLC fingerprint analysis. In the HPLC fingerprint at an absorbance of 254 nm, the peaks corresponding to ferulic acid, calycosin, formononetin, and ligustilide in DBT, RA, and RAS are indicated by arrowheads. The details of the marker identification were described previously [8]. These chemical characterizations are used to identify the standardized extracts for all the biochemical analyses. Typical fingerprints are shown.

DBT, composed of RA and RAS in a weight ratio of 5 : 1, was prepared according to the optimized extraction conditions as described previously [8]. In order to standardize the herbal extract chemically, we generated HPLC fingerprints: these fingerprints were required as to ensure the chemical composition of DBT, or extracts from RA and RAS, in all pharmacological experiments (Figure 1). By discovering the amounts of two chemical markers in RA (calycosin and formononetin) and two others in RAS (ferulic acid and ligustilide), we were able to standardize the optimal DBT. We found that the standardized DBT should contain 0.186 mg calycosin, 0.155 mg formononetin, 0.351 mg ferulic acid and 0.204 mg ligustilide per 1 g dried weight of DBT; this was in line to our previous studies [8, 9]. In addition, a standardized extract of RA in 1 g should contain 0.088 mg calycosin and 0.142 mg formononetin, while the extract of RAS in 1 g should have 0.293 mg ferulic acid and 0.316 mg ligustilide. From the calculations of extraction efficiency, the yield of DBT, RA and RAS were in a range from 29 to 32% ± 3% (*n* = 5).

Amongst different effects of DBT in cell cultures, we decided to used cultured bone cells as the study model here; because DBT-induced bone cell differentiation has been described [8]. MG-63 cell, a human cell line that exhibits phenotypic properties of osteoblast is a common cell line used in analyzing bone formation [19]. More importantly, the comparison between MG-63 cells and primary culture of osteoblasts had been done, which showed that the two types of osteoblastic cells shared a close similarity in terms of synthesis and display of glycan structures [20].

**Figure 2:**
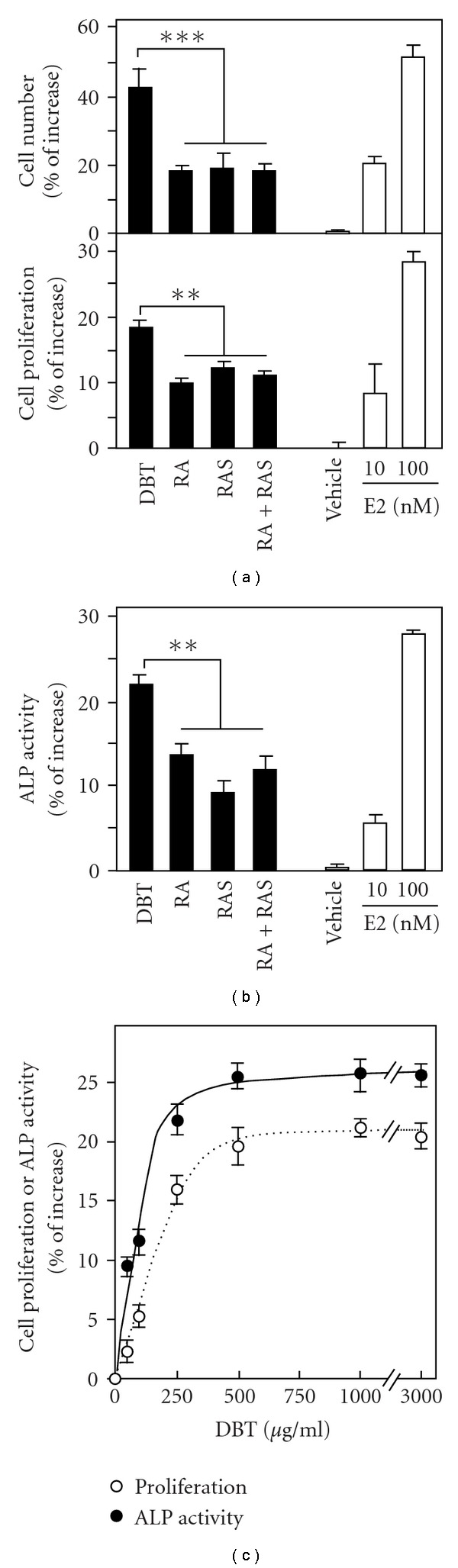
DBT increases the cell proliferation and alkaline phosphatase activity in cultured MG-63 cells. (a) Cultured MG-63 cells were treated with extracts (1 mg mL^−1^) derived from DBT, RA, RAS, and RA + RAS for 48 h to determine the cell proliferation by cell counting (upper panel) and MTT assay (lower panel). *β*-Estradiol (E2; 10 and 100 nM) was used as a positive control, while 0.0001% DMSO served as a vehicle. (b) Cultures were treated as in (a) to determine the enzymatic activity of alkaline phosphatase (ALP). (c) A dose-response curve of DBT was performed for both assays as in (a) and (b), with treatment time for 48 h. Values are expressed in percentage of increase as compared with control cultures (without herbal extract), and are in mean ± SEM, where *n* = 5, each with triplicate samples. **P < .01.

By using cell counting and MTT assay, the proliferation of MG-63 cells induced by DBT, RA, RAS, RA + RAS (boiled separately and then mixed together in 5 : 1 ratio) and estrogen were determined. As shown in Figure 2(a), DBT increased the cell number (from cell counting) and the proliferation (from MTT assay) of MG-63 cells by *∼*42% and *∼*18%, respectively, as compared with the control. This induction effect of cell proliferation was significantly higher than the effects of RA, RAS, or RA + RAS. The positive control, 17*β*-estradiol at 10 and 100 nM, caused a marked increase in the cell proliferation. The vehicle did not affect the proliferation status of MG-63 cells.

The increase of alkaline phosphatase activity in MG-63 cells occurs at the middle stage of differentiation, which could serve as an indicator of osteoblastic differentiation [21]. Similar to the effect of cell proliferation, DBT induced *∼*22% increase in alkaline phosphatase activity; this induction was significantly higher than that of RA, RAS or RA + RAS (Figure 2(b)). 17*β*-Estradiol at 10 and 100 nM caused *∼*6 and 28% increase in the enzyme activity. In the DBT-induced cell proliferation and differentiation, the effects of DBT in cultured MG-63 cells were revealed in dose-dependent manners (Figure 2(c)). Both of the assays were rather similar that 0.1 mg mL^−1^ of DBT showed an induction effect at *∼*50%.

### 3.2. Genomic Analysis of DBT-Treated Cells

**Figure 3:**
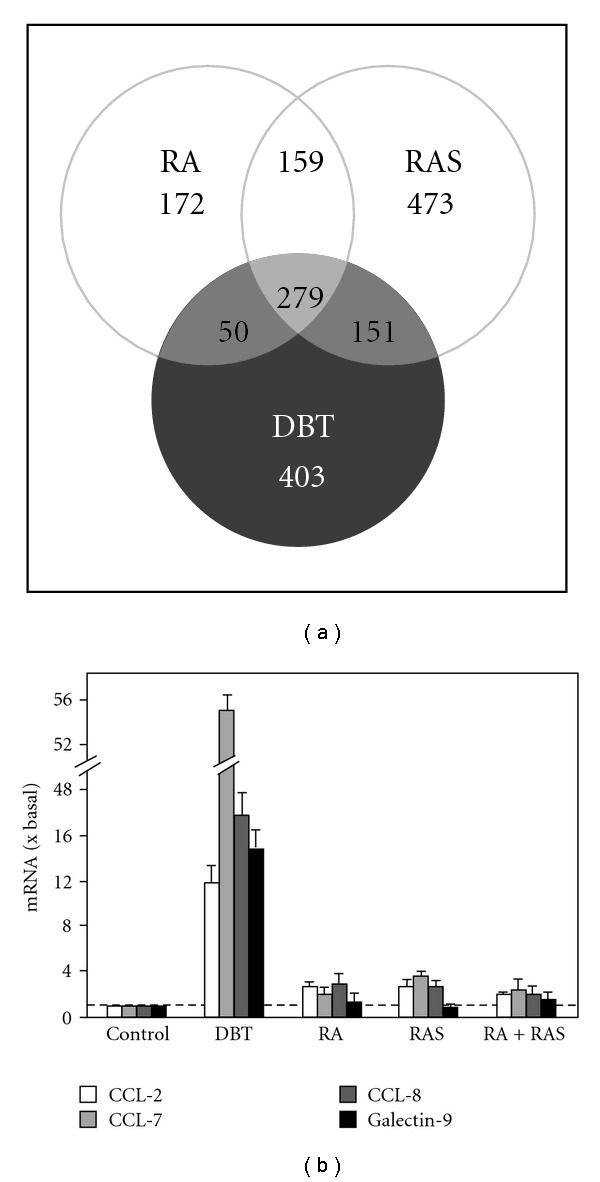
DNA microarray analysis of DBT-induced specific gene expressions and validation in cultured MG-63 cells. (a) MG-63 cells were treated with 1 mg mL^−1^ RA, RAS, or DBT for 24 h and subjected to DNA microarray analysis to determine the differential gene expressions. The DNA chip contained 7458 candidate genes and 384 reference genes: these sequences were derived from human genome. Significant changes of gene expressions were defined as regulated, either up-regulation when fluorescent signal in the sample was greater than that of control for 200%, or down-regulation when the signal was less than that for 50%. (b) Some of up-regulated genes as in (a) was validated by quantitative real-time PCR analysis. Total RNAs were extracted from cultures treated with different extracts for 24 h and used to perform real-time PCR analysis to determine the mRNA levels of CCL-2, CCL-7, CCL-8, and galectin-9. Data are normalized by ΔΔ*C*_t_ method using 18S rRNA as an internal control, and expressed as the ratio to basal reading where control (without herbal extract) equals to 1, and in mean ± SEM, where *n* = 5, each with triplicate samples.

In a Chinese herbal decoction, we are dealing with multi-components and multi-targets of the pharmacological effects. Lacking a specific biological marker and detail analysis of action mechanism are major obstacle to increase the usage of Chinese medicine. Thus, genomic approach was used here to reveal the gene expression profiling in DBT-treated MG-63 cells. The osteoblastic cell line was used here for genomic analysis instead of primary cultured osteoblasts: this was to ensure the consistence of DNA microarray results. The DNA microarray result was summarized in the supplementary table posted in the supplementary information.  Figure 3(a) shows a summary of the genomic result. In brief, there are 883 genes are regulated by DBT treatment and 403 are DBT-specific; 660 genes are regulated by RA treatment and 172 are RA-specific; 1062 genes are regulated by RAS treatment and 473 are RAS-specific. In addition, 279 genes are commonly regulated by the extracts of DBT, RA, and RAS. These numbers already provides us a snapshot that the gene activation of DBT is in distinction to that of a simple addition of RA + RAS extracts.

Amongst the 883 DBT-regulated genes, many of them are known, directly or indirectly, to play roles in bone formation (Table 1). The degree of regulation was varied among different analyzed genes; the highly regulated genes were galectin-9, CCL-8, CCL-7, and CCL-2. Thus, they were selected for validation by quantitative real-time PCR analysis. As expected from the DNA microarray results, the transcripts encoding CCL-2, CCL-7, CCL-8, and galectin-9 were markedly increased by DBT treatment (Figure 3(b)). The transcript expressions of galectin-9, CCL-2, and CCL-8 were increased by 12- to 17-folds after the challenge of DBT, while the expression level of CCL-7 mRNA was up regulated with an induction over 50-fold. In line to the result of DNA microarray analysis, the effects of DBT were significantly higher than the extracts derived from RA, RAS or even the simple mixture of RA and RAS, which suggested a strong synergistic effect in combination of RA and RAS and a crucial role of boiling the two herbs together. By comparing the results from the microarray analysis, the transcript induction by the determination of real-time PCR revealed a lower value, which could be accounted by the sensitivity of two different assays.

### 3.3. Signaling Mechanisms of DBT-Specific Gene Regulation

**Figure 4:**
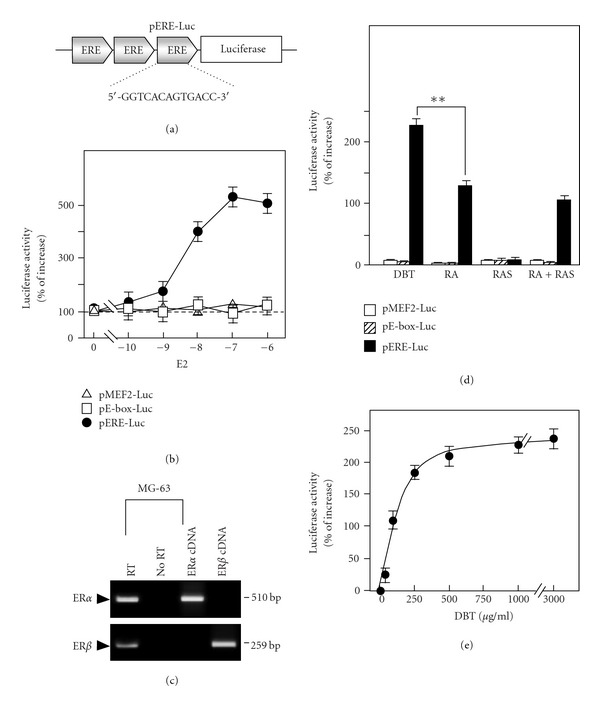
DBT induces the estrogenic effects in cultured MG-63 cells. (a) Three repeats of estrogen responsive elements (ERE: 5′-GGT CAC AGT GAC C-3′) was subcloned into a promoter-reporter vector that has a down stream reporter of firefly *luciferase* gene, namely as pERE-Luc. (b) Cultured MG-63 cells that stably transfected with pERE-Luc, or transiently transfected with pMEF2-Luc or pE-box-Luc were treated with different concentration of *β*-estradiol (E2; from 10 nM to 1 *μ*M) for 48 h to determine the transcriptional activity of pERE-Luc by luciferase assay. (c) Total RNAs were extracted from MG-63 cells to determine the presence of estrogen receptor *α* (ER*α*; 510 bp) and *β* (ER*β*; 259 bp) by RT-PCR analysis. No RT indicated the absence of contamination by genomic DNA, and ER*α* and ER*β* cDNAs served as positive control for PCR. Representative images were shown, *n* = 5. (d) Cultures were treated with extracts (1 mg mL^−1^) derived from DBT, RA, RAS and RA + RAS for 48 h to determine the luciferase activity as in (b). (e) A dose-response curve of DBT was performed as in (b). Values of the promoter-driven luciferase activities are expressed in percentage of increase as compared with control cultures (without herbal extract), and in mean ± SEM, where *n* = 5, each with triplicate samples. **P < .01.

The signaling mechanism of this DBT-induced gene expression was revealed here. DBT has been shown to activate two signaling cascades in different cell types: (i) estrogenic signal; and (ii) mitogen-activated protein (MAP) kinase signal [9, 22]. In order to test the estrogenic effects of DBT in cultured MG-63 cells, a promoter-reporter construct (pERE-Luc; see Figure 4(a)) containing three repeats of estrogen-responsive element (ERE) was stably transfected into MG-63 cells. Treatment of 17*β*-estradiol from 10 nM to 1 *μ*M produced a dose-dependent response in activating the activity of pERE-Luc in the stable transfected cell (Figure 4(b)). In contrast, no estrogenic effect was found in either pMEF2-Luc or pE-box-Luc transfected MG-63 cells. Such estrogen-mediated transcriptional activity was further confirmed by the presence of ER*α* and *β* mRNAs in cultured MG-63 cells (Figure 4(c)). No reverse transcription indicated the absence of contamination by the genomic DNA. The cDNAs encoding ER*α* and ER*β* served as positive controls for PCR. These results were consistent with the literature that both ER*α* and *β* were present in MG-63 [23, 24]. In addition, the expressions of ER*α* and *β* were not altered in our drug-treated cultures.

To test the effect of DBT, different amounts of DBT were applied onto the pERE-Luc-transfected MG-63 cells for 2 days. Application of DBT led to the activation of promoter activity by an increase of 230%: the potency was the strongest among different herbal extracts being tested (Figure 4(d)). Application of 1 mg mL^−1^ RA or 1 mg mL^−1^ RA + RAS (boiled separately and then mixed together) also induced promoter activity to *∼*110%; however, the activity was lower when compared with the activity of DBT. RAS (1 mg mL^−1^) did not have any activation effect (Figure 4(d)). Showing the specificity of DBT response, the muscle-specific gene responsive elements, MEF2 and E-box, did not respond to the challenge of DBT. In addition, the DBT treatment showed a dose-dependent response in activating pERE-driven luciferase activity (Figure 4(e)). This estrogenic effect of DBT has been shown previously in cultured MCF-7 cells (a breast cancer cell line) and the potency is comparable [9], which indicated the possession of estrogenic property of DBT in cultured MG-63 cells.

**Figure 5:**
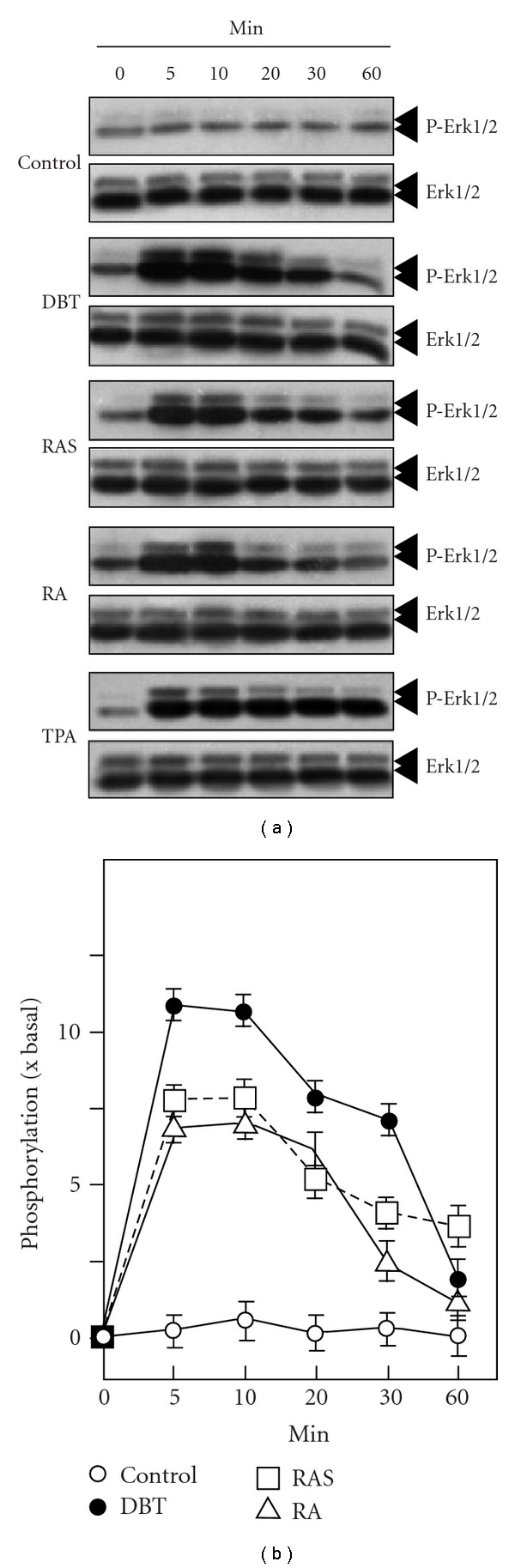
DBT induces Erk1/2 phosphorylation in cultured MG-63 cells. MG-63 cultures were serum starved for 3 h before the addition of DBT, RA, RAS, and RA + RAS extracts (1 mg mL^−1^) for different time. Total and phosphorylated inhibitor Erk1/2 and P-Erk1/2 were revealed by western blot analysis using specific antibodies. TPA at 0.1 *μ*M served as a control. The lower panel shows the quantitation of phosphorylation from the blots by calibrating the densitometry. Values are expressed as the ratio to basal reading where time 0 equals to 1, and in mean ± SEM, where *n* = 4.

MAP kinases are involved in numerous cellular responses including cell growth and differentiation, and they have been shown to participate in estrogenic effects. Therefore, we studied the phosphorylation of Erk1/2, a MAP kinase with an important role in the classical Raf-MEK-Erk pathway. The serum-starved MG-63 cultures were treated with different drugs and collected at different times. The phosphorylations of Erk1 (P-Erk1; *∼*44 kDa) and Erk2 (P-Erk2; *∼*42 kDa) were markedly increased by the addition of DBT; the activation was transient and peaked at over 10-fold in 5–10 min after the treatment (Figure 5). In comparison to DBT, the phosphorylation of Erk1/2 was also increased by *∼*7-fold when MG-63 cells were treated with RA or RAS (Figure 5). As a control, the application of TPA, a known activator of Erk1/2, induced the phosphorylation of Erk1/2 by *∼*10-fold in a sustained manner.

**Figure 6:**
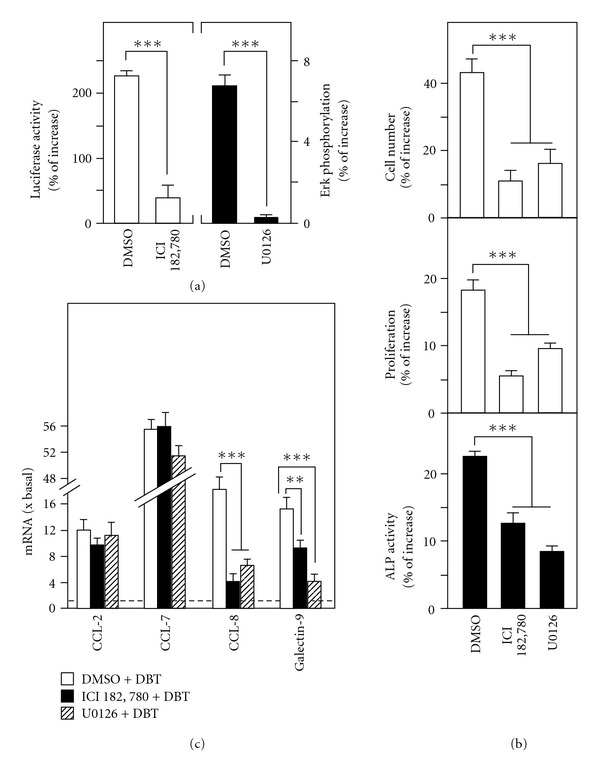
The DBT-induced osteogenic effects and gene expressions are blocked by specific inhibitors. (a) MG-63 cultures stably transfected with pERE-Luc were pre-treated with buffer (0.1% DMSO; control), ICI 182 780 (an ER blocker; 0.1 *μ*M) and U0126 (Erk inhibitor; 10 *μ*M) for 3 h before the addition of DBT (1 mg mL^−1^) for 24 h to determine the luciferase activity driven by ERE activation (left panel) and Erk1/2 phosphorylation at 5 min (right panel). (b) Cultures were treated for 48 h as in (a) to determine cell number (by cell counting), cell proliferation (by MTT assay) and alkaline phosphatase (ALP) activity. (c) To investigate the signaling mechanisms of DBT-induced gene expressions, MG-63 cells were pre-treated with inhibitors and then DBT as in (a) for 24 h to measure the change of CCL-2, CCL-7, CCL-8, and galectin-9 mRNA expressions by real-time PCR analysis. Values are expressed in percentage of increase as compared with control cultures (without herbal extract), and in the ratio to basal reading where control (without herbal extract) equals to 1, mean ± SEM, where *n* = 5, each with triplicate samples. **P < .01, ***P < .001.

To distinguish the role of estrogenic and MAP kinase signalings on DBT-induced effects in MG-63 cells, specific inhibitors for ER and MAP kinase, ICI 182 780 and U0126, respectively, were used to test the regulatory effects of DBT. The blocking effects by these inhibitors were shown in Figure 6(a). Cultures were pre-treated with inhibitors, or DMSO (control), for 3 h before the application of DBT (1 mg mL^−1^) for 2 days. The DBT-induced pERE-Luc transcriptional activity was decreased by the pre-treatment of ER blocker ICI 182 780. Besides, the DBT-induced Erk1/2 phosphorylation was reduced by U0126 application (Figure 6(a)). In cultured osteoblastic cells, both of the inhibitors partially blocked the DBT-induced cell proliferation and alkaline phosphatase activity (Figure 6(b)). These pharmacological studies suggested that the activation effects of DBT could be mediated, at least, by two signaling pathways: ER-dependent and Erk-dependent, which could be partially accounted for the regulatory mechanisms of DBT in triggering the proliferation and differentiation of osteoblastic cells.

Moreover, the roles of these inhibitors in DBT-regulated genes were also investigated by real-time PCR analysis. Application of the two inhibitors blocked the DBT-induced mRNA expressions in different extent; the blockage of CCL-8 was significant with more than 75% (Figure 6(c)). For galectin-9, the suppression effect of U0126 was greater than ICI 182 780. On the contrary, the DBT-induced CCL-2 and CCL-7 genes were totally unaffected by the two inhibitors (Figure 6(c)). These results suggest the diversity of the DBT-induced downstream signaling in cultured osteoblasts, that is, gene transcription is triggered via distinct signaling cascades.

### 3.4. Osteogenic Effects of DBT in Rat Osteoblasts

**Figure 7:**
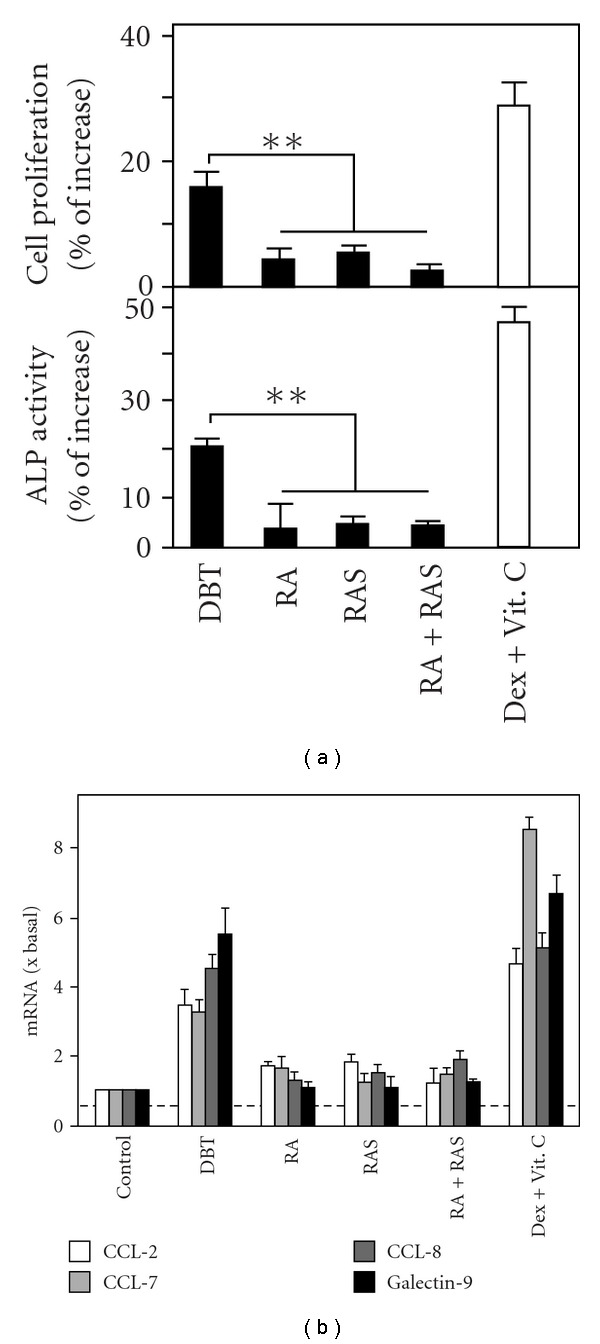
DBT stimulates the osteogenic differentiation of cultured primary osteoblasts. (a) Primary culture of osteoblasts were treated with 1 mg mL^−1^ DBT, RA, RAS, RA + RAS, and Dex + Vitamin C (positive control). After 96-h incubation, cultures were collected to perform cell proliferation and alkaline phosphatase activity assays as in Figure 2. Values are expressed in percentage of increase as compared with control cultures (without herbal extract), and are in mean ± SEM, where *n* = 5, each with triplicate samples. **P < .01. (b) Primary osteoblasts were treated as in (a) for 12 h. Total RNAs were collected to determine the change of CCL-2, CCL-7, CCL-8, and galectin-9 mRNA expressions by real-time PCR analysis as in Figure 3. Data are normalized by ΔΔ*C*_t_ method using 18S rRNA as an internal control, and expressed as the ratio to basal reading where the control (without herbal extract) equals to 1, and in mean ± SEM, where *n* = 5, each with triplicate samples.

To further support the beneficial roles of DBT on bone development, the primary culture of rat osteoblasts was employed here as another study model. The osteoblastic cultures were treated with different extracts for 96 h and collected to perform cell proliferation and alkaline phosphatase assays. As expected, DBT increased the cell proliferation and alkaline phosphatase activity by *∼*20% (Figure 7(a)), while the effects of RA, RAS or RA + RAS were all <10%. Dexamethasone and vitamin C (Dex + Vitamin C) served as a positive control for both assays. In addition, the specific gene transcriptions in DBT-treated cultures were determined. The results from real-time PCR analysis indicated that the four target genes, CCL-2, CCL-7, CCL-8, and galectin-9, were stimulated by DBT for at least 4-fold (Figure 7(b)). On the other hand, the change of mRNA levels in RA-, RAS- or RA + RAS-treated osteoblasts were <2-fold. These results were consistent with that of MG-63 cells (Figures 2 and 3), which greatly supported the uniqueness of DBT decoction and the beneficial effects of DBT on the osteoblastic cells.

## 4. Discussion

**Figure 8:**
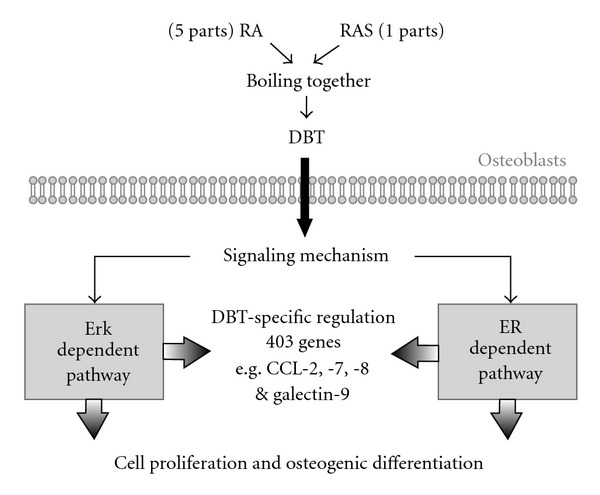
Summary for DBT-induced osteogenic effects and specific gene expressions in osteoblastic cells. Application of DBT onto osteoblastic cells triggers the downstream signaling mechanisms including the Erk-dependent and ER-dependent pathways, which may be mediated directly by DBT or indirectly by a receptor(s) on plasma membrane. Such signaling activations finally result in stimulating cell proliferation, osteogenic differentiation, and a set of specific gene transcriptions.

This study, for the first time, demonstrated the trophic roles of an ancient Chinese herbal decoction having a combination of RA and RAS in a weight ratio of 5 : 1 on bone cells. Based on our current results, a brief summary was proposed (Figure 8). According to the ancient formulation, the DBT decoction was prepared by boiling RAS and RA together in 1 : 5 mass ratio. Application of DBT onto osteoblastic cells triggered the downstream signaling cascades including the Erk-dependent and ER-dependent pathways. Such signaling activations finally resulted in stimulating cell proliferation, osteogenic differentiation and a set of DBT-regulated gene transcription. DBT possesses a better effect in stimulating cell proliferation and differentiation in cultured MG-63 cells and primary osteoblasts, as compared to that of the extracts derived from RA or RAS or RA + RAS (boiled separately and then mixed together in 5 : 1 ratio). In line to this activation effect, genomic analysis revealed a specific set of genes being regulated by DBT, but not by RA or RAS alone. These results therefore provide evidence of the uniqueness of specific combination of RA and RAS in creating the formulation of DBT. In addition, the insufficient stimulating effect of RA + RAS in cultured MG-63 cells suggests that boiling of the two herbs together is essential; this method of DBT preparation, indeed, has long been recommended by Chinese medicinal practitioners. Although the concentration of DBT in cell culture may not be relevant to that of the effective concentration in animal, the effects of DBT at mg mL^−1^ have been shown to be highly significant in different cell types [8, 9, 25]. In addition, the oral administration of DBT at a concentration of g/kg could markedly affect the oxidative status of rat [26]. Thus the biological properties of this 800-year-old decoction, as demonstrated in our previous studies and here, have been revealed in both cell culture and animal studies.

The author of DBT Li Dongyuan wrote down the formulation and the preparation methods probably based on accumulated experience in clinical application. As proposed by the author, the stimulation of “*Qi*” and “*Blood*” are the two critical effects of DBT to keep our body in a healthy balanced state. However, how this herbal decoction could be explained by modern science is not determined. We offer two hypotheses to explain the unique biological function of DBT. First, DBT might contain additional chemicals than those in the extracts of RA or RAS alone. Very likely, these additional chemicals are soluble only in DBT, that is, the boiling of RA and RAS together enhances the solubility of the chemicals. The additional chemicals could be responsible for the distinct DBT-specific effects. Unfortunately, the chemical fingerprint of DBT in our detection method (as in Figure 1) does not show any additional chemicals as compared to that of RA and RAS. Thus, the additional chemicals, if any, could be those that are not detected by HPLC, for example, polysaccharide. The optimized ratio of the two herbs in yielding more active ingredients can be another good explanation for the DBT-specific effects. Our chemical analyses showed that higher amounts of RA-derived astragaloside IV, calycosin, formononetin, and RAS-derived ferulic acid were found in the DBT decoction [8]. Second, there could be a synergistic effect of different components in DBT; this synergistic effect is not present in the extracts of the single herbs. Unfortunately, we do not have direct evidence to test these hypotheses from our DBT experiments. However, because of the failure of the RA + RAS mixture to perform the same functions as DBT, we believe that the second hypothesis is less likely.

By using cultured MG-63 osteoblasts, the gene expression profiling, after the DBT treatment, was revealed. Such DBT-regulated gene transcription was further validated in the primary culture of rat osteoblasts. The genomic analysis can reveal not only the activation effect of DBT in stimulating the proliferation and the differentiation in osteoblasts but also a set of biological responsive markers that are specifically triggered by DBT. The specific biomarkers could help to resolve the action mechanism of DBT. For instance, galectin-9, a *β*-galactoside-binding protein, expresses in various tissues that has implication in modulating cell-cell and cell-matrix interaction [27]. In addition, the role of galectin-9 in bone development was revealed to induce osteoblast proliferation through the c-Src/Erk signaling pathway [28]. CCL-2, CCL-7, and CCL-8 also called monocyte chemotactic protein 1 (MCP-1), MCP-3, and MCP-2, respectively, are specifically activated in osteoblast by DBT: they are important chemokines that belong to the CC chemokine super-family and play a critical role in the recruitment and activation of leukocytes [29, 30]. Because leukocytes produce factors capable of modulating the activities of osteoclast and osteoblast; their recruitment is representing a significant event in regulating osseous metabolism.

The regulatory effects of estrogen in MG-63 osteoblast-like cells were demonstrated previously [31]. By using anti-sense method, estrogen was shown to increase the expressions of collagen and alkaline phosphatase via an ER*β*. In line to the anti-sense study, the matrix secretion and cell proliferation of cultured bone cells were abolished in the absence of ER*β*. In addition, ER*α* is also proposed to have a role in bone development since both ER*α* and ER*β* are expressed in MG-63 cells, and ER*α* has been shown to play a vital role in mediating the osteogenic activities [32]. Therefore, these different lines of evidence further supported the close relationship between estrogen and MG-63 cell proliferation. However, the estrogenic effect in MG-63 cells is very different to that of DBT. These differences include a distinct gene profiling in gene chip analyses between estrogen and DBT and the insignificant changes of galectin-8, CCL-2, CCL-7, and CCL-8 after estrogen application in MG-63 cell cultures (data not shown). Although the ER blocker ICI 182 780 shows partial blockage of DBT-induced osteogenic effects, this result still supports the estrogenic role of DBT, because the dose of ICI 182 780 used is expected to have only *∼*20% inhibition on the possible ER binding. Moreover, our preliminary studies by using inhibitors for MAP kinase suggest that the DBT-induced gene activations could be mediated by different signaling mechanisms. This observation is in accordance with the possibilities of having multi-targets of the pharmacological properties of Chinese herbal decoction.

It is well known that steroid hormones, especially estrogen, can induce the cell proliferation and subsequently leads to an increase risk of developing breast cancer. Indeed, this issue is a major concern of estrogen replacement therapy for the menopausal women. In contrast, DBT could be developed as alternative medicines for the patients. The reasons for this notion include: (i) DBT has been used over 800 years in China, which has been proven to be safe for human; (ii) DBT does not alter the proliferation of the breast cancer cells, even at higher concentration [8, 9]; (iii) DBT improves biochemical and physiological responses, both *in vitro* [9, 22] and *in vivo* [26, 33, 34] that are related to menopausal women. In developing DBT as drug for menopausal women, here we provide the essential elements in searching the chemical and biological markers for this decoction, that is, to have a well-controlled herbal decoction for drug development.

## Figures and Tables

**Table 1:** Genes related to bone development are up regulated by DBT.

Regulated gene^a^	Symbol	Genbank no.	Fold of change^b^		
			DBT	RAS	RA
Lectin, galactoside-binding, soluble, 9 (Galectin-9)	LGALS9/Gal-9	AA434102	441.76	1.60	1.77
Small inducible cytokine subfamily A, member 8	SCYA8/CCL-8	AI268937	184.65	2.73	2.89
Small inducible cytokine A7	SCYA7/CCL-7	AA040170	175.02	0.33	6.96
Small inducible cytokine A2	SCYA2/CCL-2	AA425102	88.22	2.35	6.87
Collagen, type XVIII, *α*1	COL18*α*1	N81029	4.49	5.03	3.99
Matrix metalloproteinase 9	MMP9	T72581	3.87	5.78	7.15
Collagen, type X, *α*1	COL10*α*1	AI828306	3.44	4.09	13.95
Transforming growth factor, *β*2	TGF*β*2	AA233738	2.60	3.40	1.77
Insulin-like growth factor 1	IGF1	AA456321	2.47	—	—
Bone morphogenetic protein 1	BMP1	R56774	2.29	2.88	6.66
Epidermal growth factor	EGF	AI480081	2.00	1.19	—
Integrin, *α*2	ITG*α*2	AA463257	1.87	0.75	0.85
Msh (*Drosophila*) homeo box homolog 2	MSX2	AA195636	1.86	1.14	0.82
Collagen, type VII, *α*1	COL7*α*1	AA598507	1.86	3.49	1.76
Bone morphogenetic protein 6	BMP6	AA424833	1.73	2.28	1.27
Msh (*Drosophila)* homeo box homolog 1	MSX1	AA464197	1.66	1.45	1.28
Fibroblast growth factor receptor 3	FGFR3	AA419620	1.65	0.97	0.82
Cartilage oligomeric matrix protein	COMP	N94385	1.64	1.30	4.82
Transforming growth factor, *β*1	TGF*β*1	R36467	1.56	1.94	2.00
Bone morphogenetic protein 8	BMP8	AA779480	1.41	1.73	1.30
Bone morphogenetic protein 7	BMP7	W73473	1.39	1.75	1.64
Fibroblast growth factor receptor 1	FGFR1	R54846	1.36	1.17	1.25
Multiple inositol polyphosphate phosphatase 1	MINPP1	AA161161	0.29	0.47	0.57

^a^Genes known to play role in bone development are selected for illustration; ^b^Change of gene expressions as compared to the control (no drug treatment). “—” below detection.
